# Investigation of the role of Cremophor RH 40 and Cremophor EL in the inhibition of efflux pump of *Pseudomonas aeruginosa*

**DOI:** 10.1016/j.heliyon.2024.e33749

**Published:** 2024-06-27

**Authors:** Muhammad Asim, Yasin Ahmad, Momin Khan, Zeeshan Ahmad, Awais Khalid, Pervaiz Ahmad, Abdulhameed Khan, Fakhrul Ahsan, Mohsin Kazi, Samer H. Zyoud

**Affiliations:** a Institute of Basic Medical Sciences, Khyber Medical University, Peshawar, Pakistan; b Sarhad Institute of Allied Health Sciences, Sarhad University of Science and Information Technology, Peshawar, Pakistan; c Institute of Pathology and Diagnostic Medicine, Khyber Medical University, Peshawar, Pakistan; d Department of Microbiology, Hazara University Mansehra, Khyber Pakhtunkhwa, 21300, Pakistan; e Department of Physics, Hazara University Mansehra, Khyber Pakhtunkhwa, 21300, Pakistan; f Department of Physics, University of Azad Jammu and Kashmir, Muzaffarabad, 13100, Pakistan; g Department of Biotechnology, University of AJK, Muzaffarabad, Pakistan; h Department of Pharmaceutical and Biomedical Sciences, California Northstate University College of Pharmacy, Elk Grove, CA, 95757, USA; i Department of Pharmaceutics, College of Pharmacy, POBOX- 2457, King Saud University, Riyadh, 11451, Kingdom of Saudi Arabia; j Department of Mathematics and Sciences, Ajman University, P.O. Box 346, Ajman, United Arab Emirates

**Keywords:** Cremophor EL, Cremophor RH40, Efflux pump inhibitors, Ethidium bromide, Multidrug resistance, *Pseudomonas aeruginosa*

## Abstract

**Background:**

There is increasing emphasis on restoring the efficacy of existing antibiotics instead of developing new ones.

**Objectives:**

This study aimed to determine the role of Cremophor EL and Cremophor RH40 in the inhibition of efflux pumps in MDR *Pseudomonas aeruginosa* strains.

**Methods:**

Efflux pump-active MDR strains of *P. aeruginosa* were identified and confirmed by flow cytometry. The identified efflux-active strains were further subjected to determination of the MIC of ciprofloxacin and the synergistic role of non-ionic surfactants (Cremophor EL and Cremophor RH40) along with ciprofloxacin.

**Results:**

Out of 30 samples, 6 strains displayed high efflux pump activity. Both Cremophor EL and Cremophor RH40 showed efflux pump inhibitory roles. A 4-fold reduction in the MIC values of ciprofloxacin was observed when Cremophor EL was used along with ciprofloxacin, while a 6-fold reduction was observed when Cremophor RH40 was used along with ciprofloxacin. Both compounds showed synergistic effects with ciprofloxacin, ticarcillin and meropenem when used in a 24-well plate efflux pump inhibitory assay.

**Conclusion:**

The inhibition of the efflux pump of MDR *Pseudomonas aeruginosa* by non-ionic surfactants, namely, Cremophor RH40 and Cremophor EL, provided the best strategy to restore the efficacy of ciprofloxacin.

## 1 Introduction

Bacteria develop resistance against antibiotics through different mechanisms, such as the production of different antibiotic-degrading enzymes and changes in their genetic material called mutations [1]. Among these mechanisms, efflux pumps also play important roles in antibiotic resistance [2]. Efflux pumps are very important for bacteria to cause infection and to develop biofilms. The key important role of efflux pumps is that they are involved in the multidrug resistance phenotype (Piddock, 2006).

For the first time in history, the World Health Organization issued a global priority list at the beginning of 2017 that included bacteria that were highly resistant to antibiotics. This important list included the 12 pathogenic bacteria that were highly pathogenic to human health. This list was important to help prioritize research at both the academic and industrial levels and to make the scientific community aware of the development of new antimicrobial agents. The document also raised the issue and warned about the emergence and quick spread of gram-negative bacteria that were multidrug resistant. Among the 12 pathogens, *Pseudomonas aeruginosa* was considered one of the pathogenic bacteria. Because of its ability to produce resistance against carbapenems, this bacterium was placed on a critical priority list [3].

The efflux system of *Pseudomonas aeruginosa* expels antibiotics along with other substances from the cell using three proteins: cytoplasmic transporter proteins that utilize energy as a proton motive force, an outer membrane porin also called *OprM* and a periplasmic connective protein. The periplasmic connective protein and cytoplasmic transporter protein are collectively called multidrug efflux (*Mex*) [4].

There are many members of the efflux pump system present in microorganisms. These include the multidrug and toxic compound extrusion (MATE) family [5], the ATP-binding cassette (ABC) family [6], the major facilitator superfamily (MFS) [7], the small MDR (SMR) family [8], and the resistance nodulation cell division (RND) family. All of these families involved in antibiotic resistance extrude drugs from inside the cell to the outside, thus limiting the intracellular accumulation of antibiotics. The most important family among these is the RND family, which is involved in the transportation of multiple antibiotics and is present in gram-negative bacteria such as *Escherichia coli*, *Pseudomonas aeruginosa* and various other gram-negative pathogens [9,10].

Different studies showed that upon the use of efflux pump inhibitors in combination with antibiotics, the antibiotics exhibited better activity against resistant pathogens, which means that efflux pumps play an important role in antibiotic resistance and that efflux pump inhibitors have the ability to restore the efficacy of different antibiotics [11]. The most widely used efflux pump inhibitor against *Pseudomonas aeruginosa* are the group of peptidomimetic compounds with phenylalanine arginyl β-naphthylamide (PAβN) as a most leading compound while many other derivatives were also used in different studies based on their structure relationship [12]. Another molecule called verapamil also shown activity against efflux pump as verapamil also acts as an ion channel blocker and is widely used for the treatment of hypertension. Verapamil used in different studies for its efflux pump inhibitory activity like in *Mycobacterium tuberculosis* one of the study shown that verapamil restore the activity of bedaquiline and ofloxacin [13].

Cremophor EL and RH 40 have been used in combination with various drugs to enhance their efficacy. Non-ionic surfactants like Cremophor EL are generally more hydrophobic and less toxic to biological membranes, making them effective in dissolving water-insoluble drugs. Cremophor EL is a crucial excipient that enhances the solubility and bioavailability of poorly soluble drugs like saquinavir and paclitaxel. It is used in self-emulsifying drug delivery systems (SEDDS) alongside other excipients, such as d-α-tocopheryl polyethylene glycol succinate 1000 (TPGS), to inhibit presystemic drug metabolism and P-glycoprotein-mediated intestinal efflux, leading to increased oral absorption of cytotoxic drugs [14].

In view of these facts, the present study also used the non-ionic surfactants Cremophor EL and Cremophor RH40 as efflux pump inhibitors in combination with ciprofloxacin against MDR *Pseudomonas aeruginosa* for the restoration of ciprofloxacin efficacy.

## 2 Material and methods

This experimental study was conducted at the Department of Microbiology, Institute of Basic Medical Sciences (IBMS), Khyber Medical University (KMU), Peshawar. A total of 30 nonduplicate multidrug-resistant (MDR) *Pseudomonas aeruginosa* clinical strains were collected from August 2019 to December 2019. The transportation of samples was ensured according to proper guidelines for transporting biological specimens.

### 2.1 Chemicals and reagents

Nutrient agar, MacConkey agar, Muller Hinton agar (MHA), nutrient broth, trypticase soy broth (TSB), triple sugar iron (TSI), gram stain, oxidase reagent, catalase reagent, ethidium bromide, ethanol, ciprofloxacin, Cremophor EL and Cremophor RH40 were provided by the Department of Microbiology IBMS, KMU.

### 2.2 Identification of MDR *Pseudomonas aeruginosa*

Identification of *Pseudomonas aeruginosa* was performed on the basis of colony morphology, Gram staining and biochemical tests*.* Strains with MDR phenotypes were identified through the Kirby-Bauer disk diffusion method. Different antibiotic discs were used against *Pseudomonas aeruginosa* as recommended by CLSI, 2019. The strains of *Pseudomonas aeruginosa* that exhibited resistance to three or more different classes of antibiotics were considered MDR strains.

### 2.3 Efflux pump assay

After confirmation of MDR *Pseudomonas aeruginosa,* the strains were screened for efflux pump activity through a simple and cost-effective method called the Ethidium Bromide Agar Cartwheel Method. Trypticase soy agar plates (TSA) were prepared with varying concentrations of ethidium bromide ranging from 0.5 to 2.5 mg/l. The plates were then inoculated with a swab in a cartwheel position. Swabbing was performed from the center of the plate towards the edge of the plate. On a single plate, 8 different strains were swabbed, including the control ATCC strain of *Pseudomonas aeruginosa,* and incubated at 37 °C for 18 h.

After incubation, the plates were placed in a UV transilluminator for the measurement of fluorescence. Based upon the degree of fluorescence, three different categories were considered in the present study. Strains that showed fluorescence were considered inactive efflux pump strains, while less fluorescence was considered moderate efflux pump activity. No fluorescence was considered to indicate highly active efflux pump strains.

### 2.4 Temperature effect on efflux pump activity

To determine the effect of temperature on the activity of the efflux pump of *Pseudomonas aeruginosa*, sets of plates were placed at different temperatures after recording the initial fluorescence results. One plate was placed at 4 °C for 24 h, while the second plate swabbed with the same strain of *P. aeruginosa* was placed in an incubator for an additional 24 h at 37 °C. After incubation, the plates were again observed for fluorescence under a UV transilluminator.

### 2.5 Efflux pump inhibitory assay

The efflux pump inhibitory assay was performed to check the efflux pump inhibitory activity of Cremophor EL and Cremophor RH40. For this assay, solutions of both compounds were prepared at a 0.5 % concentration in water. After preparation of these compounds, ethidium bromide along with the compounds was added to TSA medium. One plate contained only EtBr. The second plate contained EtBr plus Cremophor EL, and the third plate contained EtBr plus Cremophor RH40.

After preparation of plates, the plates were swabbed with the cultures in a cartwheel pattern and then incubated for 18 h at 37 °C. After incubation, the results were recorded under a UV transilluminator.

### 2.6 Flow cytometry

Flow cytometry was employed to evaluate the efflux pump inhibitory activity of cremophor EL and cremophor RH40 by measuring fluorescence intensity within bacterial cells using the Moxi Flow™ instrument (Orflo technologies). The procedure began with an overnight culture of a *Pseudomonas aeruginosa* strain highly expressing efflux pumps in nutrient broth. After normalizing the bacterial count to a 0.5 McFarland turbidity standard, ethidium bromide was added at an appropriate concentration and incubated in the dark for 1 h to allow accumulation within the cells. The culture was then divided into three 1.5 mL aliquots i.e. [1] EtBr + active strain cells + glucose [2], EtBr + active strain cells + glucose + Cremophor EL, and [3] EtBr + active strain cells + glucose + Cremophor RH40), centrifuged, and washed thrice with phosphate-buffered saline (PBS). The pellets were resuspended in PBS, with glucose added to one tube to energize the cells and initiate efflux pump activity, glucose plus cremophor EL added to the second tube, and glucose plus cremophor RH40 added to the third tube. Finally, the samples were run on the Moxi Flow instrument, and the results were recorded to assess the impact of the compounds on efflux pump activity based on fluorescence measurements.

### 2.7 Determination of minimum inhibitory concentration (MIC)

The minimum inhibitory concentration (MIC) of ciprofloxacin was determined against highly active efflux pump strains of *Pseudomonas aeruginosa* using the broth microdilution method. A 96-well microtiter plate was employed, with different concentrations of ciprofloxacin ranging from 0.125 to 64 μg/mL prepared in doubling dilutions (e.g., 0.125, 0.25, 0.5, 1, 2, 4, 8, 16, 32, and 64 μg/mL). The bacterial strains were inoculated into each well containing the respective ciprofloxacin concentrations, and the plates were incubated at 37 °C for 24 h. After incubation, the MIC values were determined and interpreted according to the guidelines provided by the Clinical and Laboratory Standards Institute (CLSI, 2019).

### 2.8 Investigating the role of Cremophor EL and Cremophor RH40 in the reversal of MIC values

To investigate the role of Cremophor EL and RH40, highly resistant strains were selected with high MIC values and active efflux pump activity. The experiment was run in triplicate in a 96-well plate. One row of a 96-well plate contained a sample with ciprofloxacin at the same concentration used in MIC determination. The second row contained the sample plus ciprofloxacin and Cremophor EL. The third row contains the sample plus ciprofloxacin and Cremophor RH40. The plate was incubated at 37 °C for 18 h. After incubation, the results were visually observed for the reversal of MIC values.

### 2.9 Twenty-four well plate efflux pump inhibitory assay

A fresh culture was prepared, and the turbidity was compared with the 0.5 McFarland standard. One millilitre of Muller Hinton Broth (MHB) was distributed in all wells of the plate. Antibiotic discs to which *Pseudomonas aeruginosa* showed resistance were distributed in all wells. Colistin was used as a positive control. Then, approximately 100 μl of culture was dispensed in all wells of the plate. One well contained only MH broth as a control, while one well contained a growth control. The plate was then incubated at 37 °C for 18 h. After incubation, the difference in cell viability was checked, and colony forming units (CFUs) were calculated. The results were also visually observed.

### 2.10 Statistical analysis

One-way ANOVA was used to assess significant differences between different groups with respect to the reduction in minimum inhibitory concentrations. A p value of less than 0.05 was considered statistically significant.

## 3 Results

**Fig. 1:**
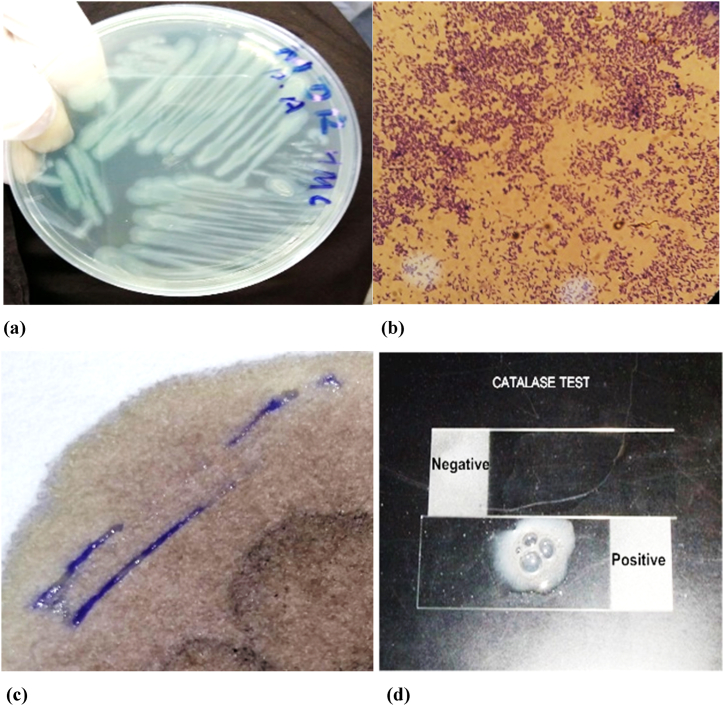
(a) Green pigment production on nutrient agar (b) Gram staining of *P. aeruginosa* (c) Oxidase test of *P. aeruginosa* (d) Catalase test of *P. aeruginosa*.

A total of 30 nonduplicate MDR *Pseudomonas aeruginosa* strains were collected from different hospital laboratories in Peshawar. The samples were again processed in the Microbiology Department of IBMS, KMU and were confirmed as *Pseudomonas aeruginosa* through morphological characteristics and various biochemical tests, such as oxidase, catalase and TSI, as shown in Fig. 1(a–d).

### 3.1 Antibiotic susceptibility pattern of *Pseudomonas aeruginosa*

**Fig. 2:**
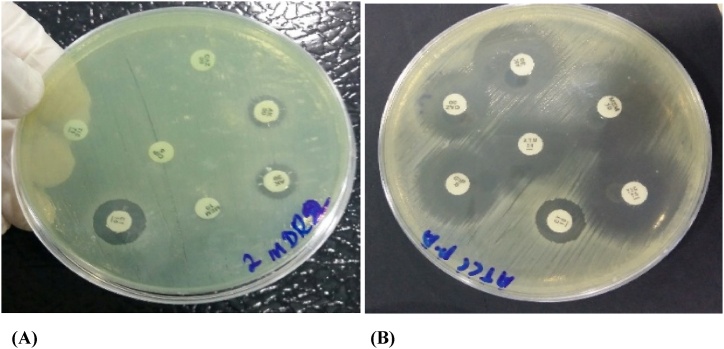
(A) The MDR strain that was resistant to more than 3 different classes of antibiotics. (B) The ATCC strain was used as a positive control and showed sensitivity to all tested antibiotics.

**Fig. 3:**
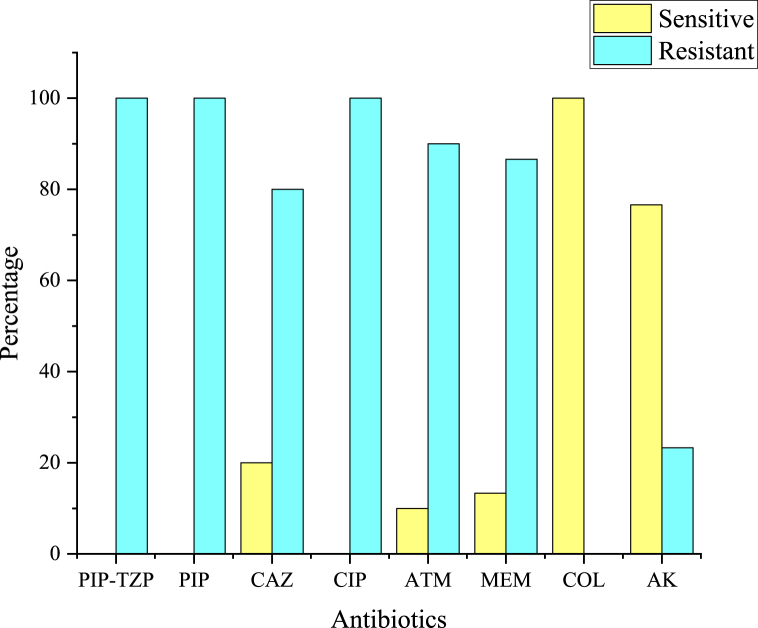
Antibiotic susceptibility profile of *Pseudomonas aeruginosa* strains used in the present study.

Out of 30 MDR strains, 100 % were found to be resistant to piperacillin, piperacillin-tazobactam, and ciprofloxacin. Six strains were sensitive to ceftazidime, four strains were sensitive to meropenem, three strains were sensitive to aztreonam, and 23 strains were sensitive to amikacin. Fig. 2A and B shows the antibiotic susceptibility results of a resistant and ATCC strain, respectively. Colistin sulfate showed 100 % activity against all 30 strains used in the present study, as depicted in Fig. 3.

### 3.2 Ethidium bromide agar cartwheel method for efflux pump activity

**Fig. 4:**
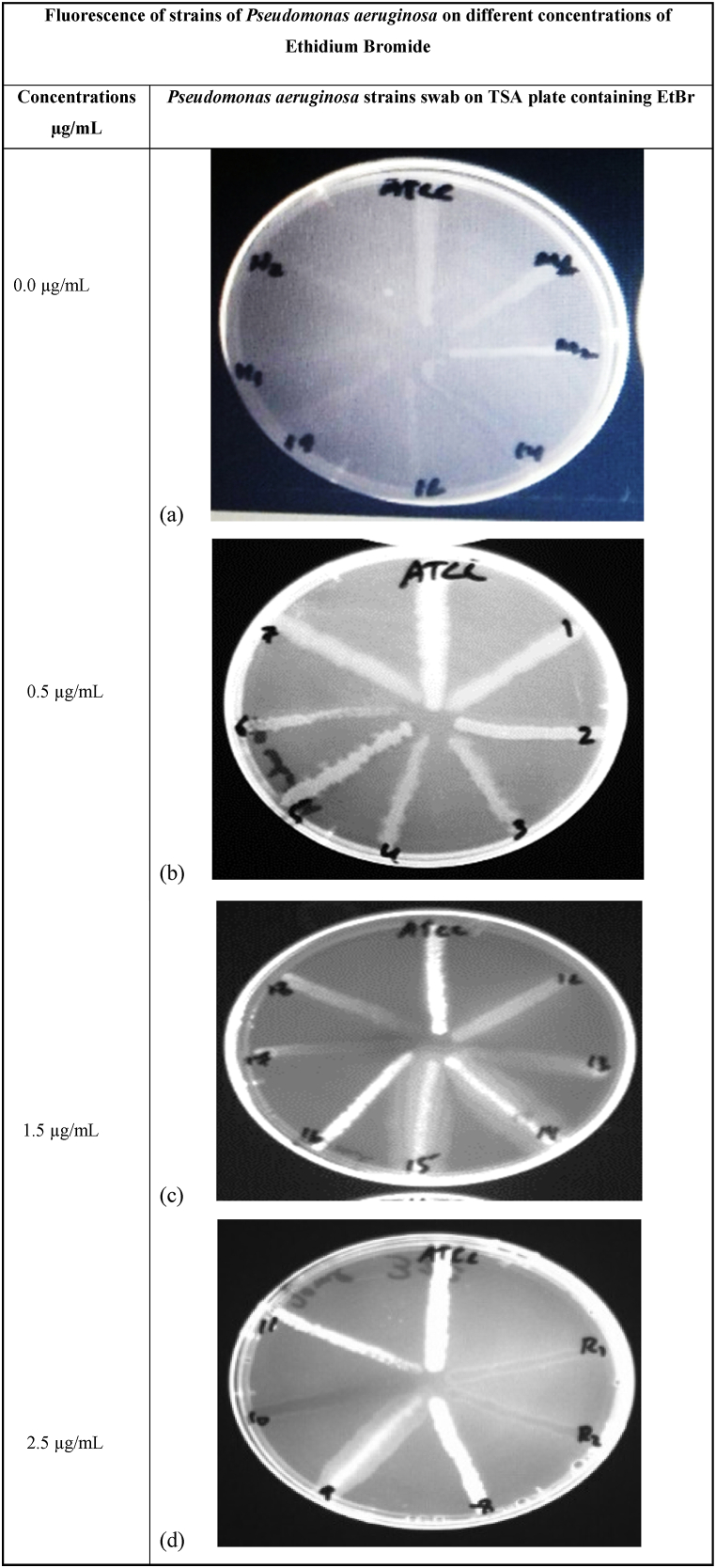
Fluorescence of *P. aeruginosa* strains cultured on TSA plates containing different concentrations of ethidium bromide (a) 0 μg/mL, (b) 0.5 μg/mL, (c) 1.5 μg/mL and (d) 2.5 μg/mL to check efflux pump activity.

**Fig. 5:**
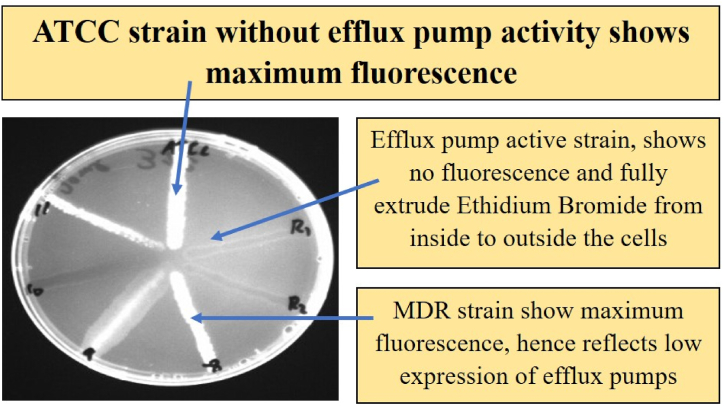
Plates containing different strains of *Pseudomonas aeruginosa,* including active efflux pump strains, showed no fluorescence, while inactive strains showed high fluorescence. The ATCC strain was used as a control.

Out of 30 strains, 6 strains were identified with highly expressed efflux pumps because they showed no fluorescence at concentrations as high as 2.5 mg/l, while 19 strains were found to have moderate efflux pump activity at a higher concentration (2.5 mg/l), and the efflux activity was high at 1.5 mg/l EtBr, as shown in Fig. 4. Five strains were found without efflux pump activity because they were unable to extrude the lower concentration of EtBr, as shown in Fig. 5.

### 3.3 Temperature effect on the efflux pump system

**Fig. 6:**
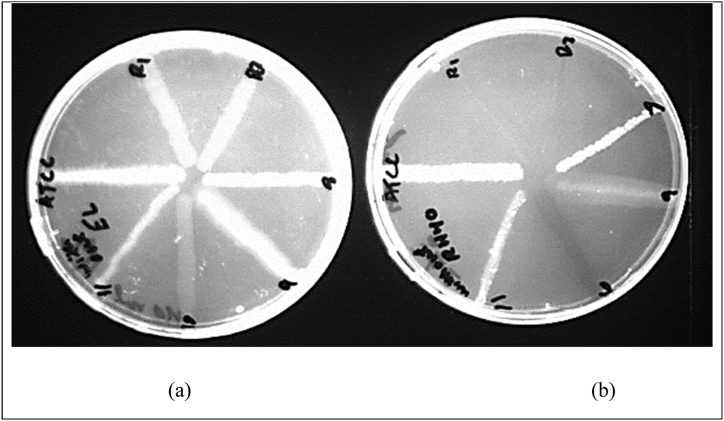
(A) The plate was placed for an extra 24 h in a refrigerator at 4 °C and regained fluorescence. (B) Plate placed for an extra 24 h in an incubator at 37 °C; cells of *Pseudomonas aeruginosa* fully extruded EtBr. No fluorescence was detected in active efflux pump strains.

After extra incubation of plates at 4 °C and 37 °C in an incubator, the plate that was placed in a refrigerator regained fluorescence, while the plate that was placed in an incubator showed more efflux pump activity and extruded all EtBr from inside the cells, as shown in Fig. 6(a–b).

### 3.4 Efflux pump inhibitory assay

*P. aeruginosa* strains cultured on media plates containing EtBr showed no fluorescence when observed under a UV transilluminator due to the highly active efflux pump system.

**Fig. 7:**
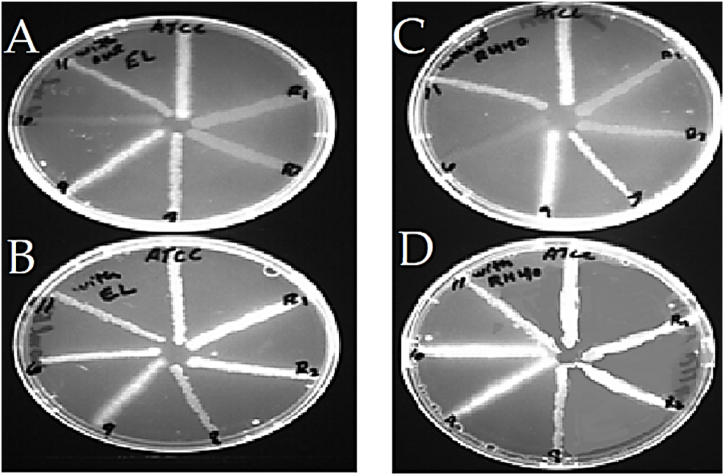
(a) The plate does not contain Cremophor EL, so the efflux-active strains extrude EtBr from inside the cells to outside. (b) The plate below contains Cremophor EL, which blocked the extrusion so that active efflux pump strains start fluorescence. (c) The plate does not contain Cremophor RH40, so cells extruded EtBr. (d). The plate containing compound RH40 blocked the efflux pump, so the cells became fluorescent.

On plates that contained ethidium bromide, Cremophor EL and Cremophor RH 40, the efflux pump-active strains showed high fluorescence in the presence of these compounds, as shown in Fig. 7(a–d).

### 3.5 Flow cytometry

**Fig. 8:**
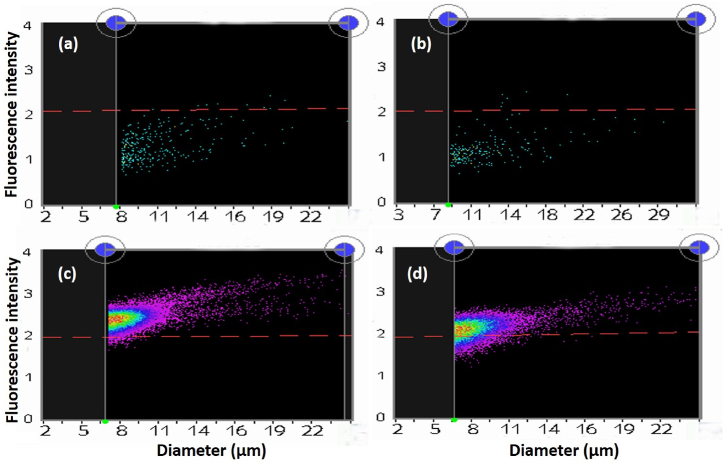
Flow cytometry images obtained for confirmation of efflux pump inhibitory activity. (a) Control sample that contains only *Pseudomonas aeruginosa* cells without EtBr and compounds in the figure. The red line differentiates between fluorescent and nonfluorescent cells. The upper population of cells was considered fluorescent cells, while the population below the red line was considered nonfluorescent cells. (b) The sample contains EtBr + cells + glucose for energizing the cells. Cells were observed without fluorescence because the cells expelled EtBr outside. **(c)** Because of the presence of Cremophor, RH40 cells were unable to expel EtBr from the cells, so the cells showed high fluorescence. (d) Due to the presence of Cremophor, EL cells were unable to extrude EtBr; hence, the cells showed high fluorescence.

For confirmation, the fluorescence intensity was also checked through a sensitive method called flow cytometry. After incubation, the samples were run on a Moxi flow cytometer. The samples that contained compounds showed greater fluorescence intensity, while the samples that contained only cells, EtBr and glucose showed only a few cells showing fluorescence, as shown in Fig. 8(a–d). A p-value of 0.04 was obtained when one-way ANOVA test was applied which mean the data is highly significant.

### 3.6 Minimum inhibitory concentration (MIC)

Ciprofloxacin was used against six active strains that showed active efflux pump activity to check their MIC. All strains were found to have high MIC values ranging from 2 μg/mL to 16 μg/mL, as shown in Table 1.

**Table 1:** MIC values of six active efflux pump strains of *Pseudomonas aeruginosa*.

Strains code	MIC μg/mL	Status Resistant/sensitive
R1	4 μg/mL	Resistant
R2	8 μg/mL	Resistant
10	16 μg/mL	Resistant
5	2 μg/mL	Resistant
7	8 μg/mL	Resistant
3	2 μg/mL	Resistant

### 3.7 Effect of Cremophor EL and RH40 on MIC values of ciprofloxacin

Among the six active efflux pump strains, one strain was selected that had a high MIC value. A strain with code 10 was selected because it showed an MIC (16 μg/mL) higher than the others and was used to check the effect of Cremophor EL and RH40 on its MIC. After incubation, the plate was visually observed, and the MIC of ciprofloxacin without the compound was 16 μg/mL, while the MIC of ciprofloxacin along with Cremophor EL was 4 μg/mL with a two-fold reduction and the MIC of ciprofloxacin in combination with Cremophor RH40 was 2 μg/mL with an 8-fold reduction in the MIC value, as shown in Table 2.

**Table 2:** Effect of Cremophor EL and Cremophor RH40 on the MIC value of ciprofloxacin.

Tested Strain code	MIC μg/mL		
	Ciprofloxacin alone	Ciprofloxacin + Cremophor EL	Ciprofloxacin + Cremophor RH40
10	16 μg/mL	4 μg/mL	2 μg/mL

### 3.8 Twenty-four well efflux pump inhibitory assay

**Fig. 9:**
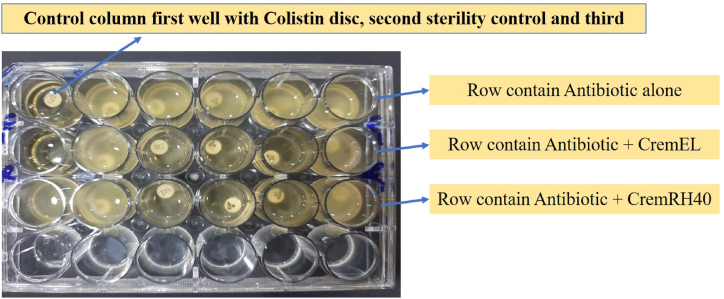
Twenty-four-well plate assay showing the synergistic effect of compounds used along with antibiotics.

Out of 5 antibiotics (ciprofloxacin, meropenem, azetronem, ceftazidime and ticarcelin), three antibiotics, i.e., ciprofloxacin, meropenem and ticarcelin, when used in combination with Cremophor EL and RH40, showed greater efficacy by reducing the growth of the resistant strain of *Pseudomonas aeruginosa* compared to the use of these antibiotics alone, as shown in Fig. 9 and Table 3.

**Table 3:** **Effect of Cremophor EL and RH40 on antibiotic resistance**.

Antibiotics	CFU(10^5^)			Efflux pump inhibitors activity
	With antibiotic alone	With antibiotic + crem EL	With antibiotic + crem RH40	Reversal/reduction
Ceftazidime	2.5 × 10^5^	2.2 × 10^5^	1.9 × 10^5^	Less reduction
Ticarcellin	2.3 × 10^5^	5.3 × 10^4^	3.9 × 10^4^	Reduction
Meropenem	2.7 × 10^5^	3.0 × 10^4^	2.8 × 10^4^	Reduction
Azetronem	2.6 × 10^5^	2.5 × 10^5^	2.5 × 10^5^	No effect
Ciprofloxacin	2.4 × 10^5^	2.7 × 10^4^	2.6 × 10^4^	Reduction

## 4 Discussion

The emergence and spread of multidrug-resistant (MDR) bacterial strains, particularly within the *Pseudomonas aeruginosa* species, pose a significant threat to public health. As the arsenal of effective antibiotics decreases, novel approaches to combat antibiotic resistance are urgently needed. This study takes a commendable step in this direction by exploring the potential of non-ionic surfactants, Cremophor EL and Cremophor RH40, to inhibit efflux pumps in MDR *P. aeruginosa* strains, thereby restoring the efficacy of ciprofloxacin.

In the present study, we demonstrated for the first time the role of Cremophor RH40 and Cremophor EL in inhibiting the efflux pump of MDR strains of *Pseudomonas aeruginosa*. The MDR strains of *Pseudomonas aeruginosa* used in the present study were able to extrude higher concentrations of EtBr, clearly showing their efflux pump activity. Ethidium bromide was used because it acts as a substrate for efflux pumps such as antibiotics. *Pseudomonas aeruginosa* strains that overexpressed efflux pumps also showed higher MIC values that exceeded the standard limit or break point set by CLSI 2019 against ciprofloxacin.

The outcomes of the study reveal intriguing findings regarding the inhibitory potential of Cremophor EL and Cremophor RH40. The reduction in ciprofloxacin's MIC values when used in conjunction with these surfactants implies a potentiation of antibiotic activity. The substantial fold reduction in MIC values demonstrates the effectiveness of these surfactants in overcoming efflux-mediated resistance. These findings are consistent with previous studies that have highlighted the potential of efflux pump inhibitors to restore the activity of antibiotics that are otherwise rendered ineffective by efflux mechanisms [[15], [16], [17]].

In the present study, a total of 30 MDR strains of *Pseudomonas aeruginosa* were screened for their efflux pump activity through a method called the ethidium bromide agar cartwheel method [18]. The study determined that the prevalence of highly efflux-active strains was 20 %, that of moderately active strains was 63.3 %, and that of strains showing no efflux pump activity was 16.6 %. A similar study was performed by Manju Suresh et al.*,* 2013 [19]. They also used the ethidium bromide agar cartwheel method for the identification of efflux pump-active strains and studied efflux pump activity among four different gram-negative bacteria, in which the most prevalent and highly active efflux pump strains were *Pseudomonas aeruginosa*. They also found that out of 23 *Pseudomonas aeruginosa* strains, 11 strains were able to extrude higher concentrations of ethidium bromide, which acts as an efflux pump substrate for the cells.

Efflux pump inhibitors have been studied by many researchers around the world. They used it as an adjuvant with antibiotics to restore the efficacy of those antibiotics to which different strains of bacteria showed resistance. The present study used two well-known inhibitors, Cremophor RH40 and Cremophor EL, that showed efflux pump inhibitory activity against P-glycoprotein in eukaryotic cells.

Casadesús et al., 2014 also used the plate method to detect the role of efflux pump inhibitors and then confirmed it through flow cytometry. They found that in the presence of an inhibitor (PAβN), the cells retained EtBr (substrate for efflux pump) and showed high fluorescence intensity compared to those cells in which inhibitors were not used [20]. Paixão et al., 2009 also used flow cytometry to measure efflux pump activity and found that in the presence of an efflux pump inhibitor, EtBr accumulation occurred, and the fluorescence intensity of the cells increased. The reason behind this is very simple: the inhibitors inhibit the activity of the efflux pump, so EtBr remains in the cells and fluoresces, while without the use of inhibitors, the cells extrude EtBr outside, and hence, no fluorescence occurs [21].

Keeping in mind the role of Cremophor RH40 and Cremophor EL in the inhibition of efflux pumps in eukaryotic cells, this study used both compounds against the efflux pump of *Pseudomonas aeruginosa* and found that both compounds have the ability to inhibit the efflux pump of *Pseudomonas aeruginosa* and helped restore the efficacy of ciprofloxacin, such as a fourfold reduction in the case of Cremophore EL and a sixfold reduction in the presence of Cremophore RH40 compared to the single use of ciprofloxacin. A study was performed by Martins et al.*,* 2011, where they used three different compounds as efflux pump inhibitors (TZ-thioridazine; CPZ-chlorpromazine; PAβN-Phe-Arg-β- napthylamide) in combination with ciprofloxacin against *E. coli,* and they found that in the presence of these inhibitors, a fourfold reduction in the MIC of ciprofloxacin occurred [22].

Takrami et al.*,* 2019 used curcamin-encapsulated nanoparticles as efflux pump inhibitors and found that when curcamin-encapsulated nanoparticles were used in combination with ciprofloxacin, they caused 50 % more deaths of clinical isolates of *Pseudomonas aeruginosa* compared to ciprofloxacin alone [23]. Ferrer et al., 2019 used polymyxin B nonopeptide (PMBN) to increase the permeabilization of *Pseudomonas aeruginosa* cells to synergize the activity of antibiotics with an efflux pump inhibitor (PAβN), and they found that increasing permeabilization for efflux pump inhibitors increased the efficacy of antibiotics and lowered the toxic effect of efflux pump inhibitors [24].

Talebi et al.*,* 2016 investigated the role of efflux pump inhibitors on the revarsal MIC value of antibiotics. In their study, they used CCCP as an efflux pump inhibitor in combination with ciprofloxacin against *Pseudomonas aeruginosa* and found a twofold-fold or higher MIC reduction [25]. A study by Matsumoto et al., 2011 investigated the role of PAβN in the inhibition of efflux pumps in *E. coli* and *Pseudomonas aeruginosa* and found that PAβN synergistically decreased the MIC of ciprofloxacin and erythromycin against *E. coli* and *Pseudomonas aeruginosa*. The main disadvantage of all these efflux pump inhibitors is their toxic effects in *in vivo* studies [26].

## 5 Conclusion

The efflux pump is the most important mechanism that highly contributes to the MDR phenotype of *Pseudomonas aeruginosa*. Therefore, efflux pump inhibitors can play an important role in overcoming this issue, as this study screened compounds for their efflux pump inhibitory activity and found that both compounds have the ability to inhibit efflux pump activity. Hence, it is concluded that nonionic surfactants (Cremophor EL and RH40) effectively inhibited the efflux pump activity of MDR *Pseudomonas aeruginosa.* These newly screened compounds can offer the best option for the restoration of ciprofloxacin efficacy against resistant *Pseudomonas aeruginosa.*

## CRediT authorship contribution statement

**Muhammad Asim:** Writing – original draft, Methodology, Investigation, Formal analysis, Data curation. **Yasin Ahmad:** Writing – review & editing, Writing – original draft, Visualization, Investigation, Formal analysis. **Momin Khan:** Writing – review & editing, Writing – original draft, Data curation, Conceptualization. **Zeeshan Ahmad:** Methodology, Investigation, Formal analysis. **Awais Khalid:** Writing – review & editing, Validation, Methodology, Investigation. **Pervaiz Ahmad:** Writing – review & editing, Software, Investigation, Funding acquisition. **Abdulhameed Khan:** Resources, Methodology, Formal analysis. **Fakhrul Ahsan:** Writing – review & editing, Methodology, Conceptualization. **Mohsin Kazi:** Writing – review & editing, Writing – original draft, Validation, Project administration. **Samer H. Zyoud:** Writing – review & editing, Validation, Methodology.

## Declaration of competing interest

The authors declare that they have no known competing financial interests or personal relationships that could have appeared to influence the work reported in this paper.
